# Exploring the Role of MRCP+ for Enhancing Detection of High-Grade Strictures in Primary Sclerosing Cholangitis

**DOI:** 10.3390/jcm14155530

**Published:** 2025-08-06

**Authors:** James Franklin, Charlotte Robinson, Carlos Ferreira, Elizabeth Shumbayawonda, Kartik Jhaveri

**Affiliations:** 1 Department of Medical Science & Public Health, Institute of Medical Imaging and Visualisation, Bournemouth University, Poole BH12 5BB, UK; 2 Radiology Department, Royal Berkshire NHS Foundation Trust, Reading RG1 5AN, UK; 3 Perspectum Ltd., Oxford OX4 2LL, UK; 4 Joint Department of Medical Imaging, University Health Network, University of Toronto, Toronto, ON M5G 2C4, Canada; 5 Faculty of Medicine, University of Toronto, Toronto, ON M5S 3K3, Canada

**Keywords:** MRCP, quantitative, assessment, evaluation support, reader confidence

## Abstract

**Background:** Identifying high-grade strictures (HGS) in patients with primary sclerosing cholangitis (PSC) relies upon subjective assessments of magnetic resonance cholangiopancreatography (MRCP). Quantitative MRCP (MRCP+) provides objective evaluation of MRCP examinations, which may help make these assessments more consistent and improve patient management and selection for intervention. We evaluated the impact of MRCP+ on clinicians’ confidence in diagnosing HGS in patients with PSC. **Methods**: Three expert abdominal radiologists independently assessed 28 patients with PSC. Radiological reads of MRCPs were performed twice, in a random order, three weeks apart, then a third time with MRCP+. HGS presence was recorded on semi-quantitative confidence scales. The cases where readers definitively agreed on presence/absence of HGS were used to assess inter- and intra-reader agreement and confidence. **Results**: When using MRCP alone, high intra-reader agreement was observed in identifying HGS within both intra- and extrahepatic ducts (64.3% and 70.8%, respectively), while inter-reader agreement was significantly lower for intrahepatic ducts (42.9%) than extrahepatic ducts (66.1%) (*p* < 0.01). Using MRCP+ in the third read significantly improved inter-reader agreement for intrahepatic HGS detection to 67.9% versus baseline reads (*p* = 0.02) and was comparable with extrahepatic ducts. Reader confidence tended to increase when supplemented with MRCP+, and inter-reader variability decreased. MRCP+ metrics had good performance in identifying HGS in both extra-hepatic (AUC:0.85) and intra-hepatic ducts (AUC:0.75). **Conclusions**: MRCP evaluation supported by quantitative metrics tended to increase individual reader confidence and reduce inter-reader variability for detecting HGS. Our results indicate that MRCP+ might help standardize MRCP assessment and subsequent management for patients with PSC.

## 1. Introduction

Primary sclerosing cholangitis (PSC) is a complex biliary disease (involving both autoimmune and other immune-related factors) characterized by slow but persistent inflammation and fibrosis surrounding the bile ducts [1]. This condition often leads to multi-focal stricturing of the intrahepatic and/or extrahepatic bile ducts, with resultant cholestasis and liver damage, although the natural history of the disease is variable [2]. Identifying clinically significant strictures in PSC is of utmost importance, as these strictures may be targets for therapeutic intervention and can be malignant in up to 15% to 20% of patients [1]. Failure to detect and manage these strictures promptly can cause significant negative impacts on patients, including potential decreased survival rates due to the undetected development of cholangiocarcinoma [3].

The accurate identification of clinically significant strictures and true disease burden in PSC remains challenging. Historically, clinical guidelines categorized strictures requiring intervention based on their endoscopic retrograde cholangiopancreatography (ERCP) caliber (i.e., <1.5 mm diameter in common bile duct [CBD] and <1 mm diameter in common hepatic duct [CHD] within 2 cm of the bifurcation at the hilum), referred to as high-grade strictures (HGS) or dominant strictures. Although ERCP is used to evaluate symptomatic patients with suspected biliary strictures, due to its invasive nature and potential for complications, it is not typically used as a first-line diagnostic tool for PSC [4]. Instead, non-invasive tests, particularly magnetic resonance cholangiopancreatography (MRCP), are used alongside other clinical assessments to evaluate the distribution and severity of disease.

Currently, the assessment of MRCP images by radiologists introduces significant variability in the detection of high-grade (dominant) strictures [5]. A study evaluating agreement on MRCP findings between radiologists and hepatologists revealed minimal concordance in identifying biliary changes or high-grade strictures (HGS) requiring intervention [6]. Such misdiagnoses of HGS can lead to unnecessary intervention, e.g., ERCP, with the associated potential morbidity and healthcare costs [7]. Therefore, as noted by the European Association for the Study of the Liver (EASL) PSC clinical guidelines, there is a need for standardization of MRCP image analysis to support identification of potentially problematic strictures [8].

Quantitative MRCP (MRCP+) is an AI-enabled software tool that builds on standard MRCP by generating quantitative models and metrics that provide detailed and accurate characterization of the biliary tree [9,10] and can support both visualization and direct assessment of ductal morphology. The EASL PSC guidelines note that MRCP+ has utility as a prognostic tool for prediction of clinical outcomes [8], and published evidence has shown MRCP+ metrics as having utility to support risk stratification in PSC [11,12]. With this growing body of evidence highlighting clinical utility, our aim was to investigate the impact of including MRCP+ as part of the decision-making process for the diagnosis of HGS compared with MRCP alone.

## 2. Methods

### 2.1. Study Design and Population

This was a cross-sectional retrospective analysis of data derived from 28 patients with PSC [13]. Patients aged over 18 years with an established PSC diagnosis were included in the study between 2016 and 2018. Alongside clinical assessment, liver biochemistry and liver stiffness assessments (using vibration-controlled transient elastography [VCTE]), patients underwent a non-contrast 3D MRCP.

Exclusion criteria for this study included those with liver cirrhosis, decompensated liver, concomitant viral hepatitis, alcohol-induced liver disease, metabolic disfunction associated liver disease (MASLD), drug-induced liver injury, secondary sclerosing cholangitis or immunoglobulin G4 (IgG4)-related disease, history of hepatobiliary malignancy, presence of biliary stents or external biliary drains, history of liver transplant assessment and procedure, absence of informed consent, or contraindications to magnetic resonance imaging (MRI).

### 2.2. Ethical Considerations

All patients gave informed consent, and the study observed the principles identified in the 1975 Declaration of Helsinki and Good Clinical Practice (GCP). Local ethical approval was received from the National Research Ethics Service, West Midlands (Ref: WM/14/0010) and was registered with the International Standard Randomised Controlled Trial Number (IRCTN) registry (ISRCTN39463479). The study was funded by an NIHR grant (project number: 15912). All participant-identifiable information was securely stored and encrypted on servers located at the study site.

### 2.3. Imaging Protocol and Post-Processing

Participants fasted for at least four hours prior to each scan on a 3 Tesla MRI Scanner (Verio, Siemens Healthineers, Erlangen, Germany). Heavily T2-weighted MRCP images were acquired using 3D multi-shot fast/turbo spin echo sequences with extended echo trains and short echo spacing to produce high-resolution 3D volumetric images. Imaging parameters included a TE of 708 ms, 72 contiguous slices, and an acquisition matrix of 258 × 320, reconstructed to 320 × 320, with a 400 × 400 mm field of view and a voxel size of 1.25 × 1.1 × 1.25 mm [9]. Scans were performed with respiratory gating using navigator tracking during the expiratory phase, resulting in variable TR depending on the patient’s breathing rate. Fat signals were suppressed using fat saturation, inversion-recovery imaging, and opposed-phase imaging techniques, and scanning time was reduced by parallel imaging techniques (parallel imaging factor: 3).

**Figure 1:**
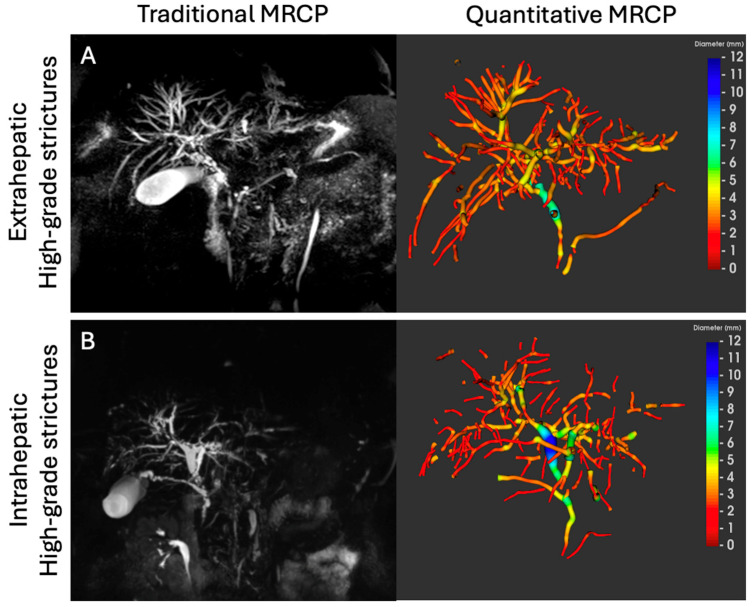
Traditional MRCP (left) and MRCP+ (right) images from a patient with PSC with (**A**) extrahepatic and (**B**) intrahepatic high-grade strictures. Quantitative MRCP (MRCP+) is an artificial intelligence-enabled software that enhances conventional MRCP to produce quantitative models with metrics that characterize the biliary tree and can support both visualization and direct assessment of ductal morphology. Warmer colors (red) are used to indicate ductal narrowing (strictures) whilst cooler colors (blue) indicate ductal widening (dilatations). Abbreviations: MRCP, magnetic resonance cholangiopancreatography; MRCP+, quantitative magnetic resonance cholangiopancreatography.

After image acquisition and de-identification, the non-contrast MRCP scans were post-processed using MRCP+ (Perspectum Ltd., Oxford, UK) to derive quantitative metrics of the biliary tree (Supplementary Table S1). As part of this process, a color-coded 3D model was generated to visualize diameter variations along each bile duct segment [9] (Figure 1).

### 2.4. Defining High-Grade Strictures

Following acquisition and de-identification of non-contrast MRCP images, but prior to reading all MRCP scans included in this study, three expert consultant hepatobiliary radiologists (all with >17 years’ individual experience each) attended an alignment session and agreed on the definition and assessment of HGS. An extrahepatic HGS was defined as being clinically relevant if they met the criteria defined by Venkatesh and colleagues [5], specifically if the minimum duct width was less than 75% of the local maximum width. Additionally, intrahepatic HGS were [5] determined relevant if there was a focal intraparenchymal stricture with associated dilatation of the intrahepatic ducts that was considered disproportionate to disease elsewhere in the liver [5].

### 2.5. Radiologist Image Evaluation

**Figure 2:**
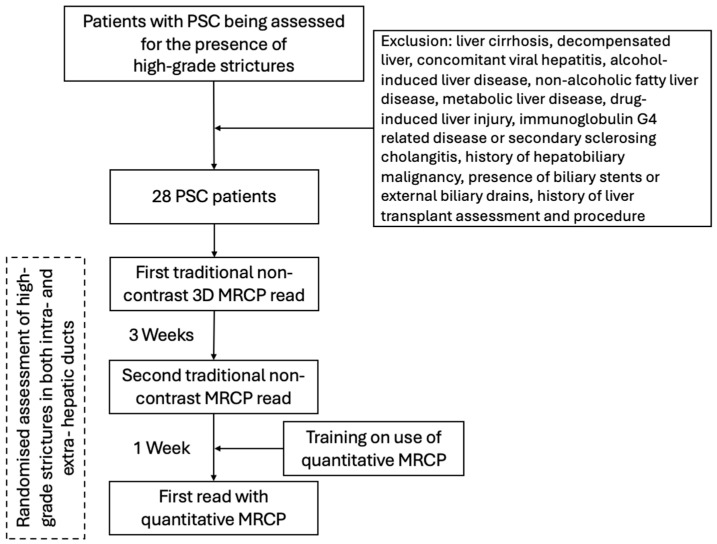
Summary of participant selection and radiologist image evaluation. Abbreviations: MRCP, magnetic resonance cholangiopancreatography; PSC, primary sclerosing cholangitis.

Following consensus agreement on the definition of HGS, the radiologists were independently provided with anonymized MRCP scans. In this study, three independent assessment sessions were undertaken by each individual radiologist (Figure 2).

To evaluate inter- and intra-reader variability, at the first assessment session, the radiologists reported the presence of HGS in intra- and extra-hepatic ducts on the MRCP images, utilizing a semi-quantitative Likert confidence scale with four levels: ‘definitely’, ‘probably’, ‘probably not’, or ‘definitely not’. To ensure that the baseline (first) read was reflective of real-world assessment and not impacted by any internal bias, radiologists were only trained on use of the MRCP+ and the structure of MRCP+ report following the first read. At the second assessment session, three weeks after the first and following the training, the radiologists performed a repeat assessment of the MRCP images (without reference to their first session assessment). The final session took place one week after the second. Here, the radiologists were provided with MRCP+ reports for the same cases (*n* = 28) and asked to evaluate the presence of HGS. To ensure unbiased evaluation, the order of the MRCP images was randomized prior to evaluation at each session. Randomization was performed by a data analyst using the study IDs and a standard randomization method; radiologists were blinded to the randomization and study IDs during all assessments.

To ensure alignment with clinical practice realities and reflect real-world conditions, radiologists’ agreement on HGS presence during the first read assessment of MRCP images was used to compare the influence of MRCP+ metrics on the assessment in the third read.

### 2.6. Sample Size Calculation

The aim of this study was to determine the impact of MRCP+ on clinicians’ confidence in diagnosing HGS in patients with PSC. The sample size was determined by a statistical power analysis. Considering the null hypothesis that an expert radiologist can correctly identify problematic strictures 80% of the time [14] and the worst-case scenario where MRCP+ can benefit 20% of patients, using an acceptable level of significance of 5% (α = 0.05) and 80% power, a minimum sample of 26 patients are required to meet the primary endpoint. As this was a retrospective study, there was no loss rate considered.

### 2.7. Statistical Data Analysis

Baseline participant characteristics were summarized using descriptive statistics. Categorical variables were presented as counts and percentages, while continuous variables were reported as means with standard deviations (±SD) or as medians with interquartile ranges (IQR), depending on data distribution.

Intra-reader and inter-reader agreement was calculated as the percentage (%) agreement between the reads for each MRCP image dataset by the same radiologist and between the reads for each MRCP image dataset by the three different radiologists, respectively. Intra-reader and inter-reader confidence in the decision was computed as the proportion of cases where radiologists agreed on the definite presence vs. absence of HGS between their three individual reads and between the reads of the other radiologists. Cohen’s Kappa coefficient (κ), positive percentage agreement (PPA), and negative percentage agreement (NPA) were used to further establish the inter-reader agreement between radiologists. For agreement levels, κ values below 0.20 were taken to suggest no, 0.21–0.40 poor, 0.41–0.60 moderate, 0.61–0.80 good, and 0.81–1.00 excellent agreement.

Various levels of agreement were recorded between the readers: full agreement (unanimous agreement between all readers), partial agreement (agreement between two readers), and disagreement (no agreement between readers). Where there was unanimous agreement between all three readers that there was either ‘definitely’ or ‘probably’ the presence of an HGS at the first read, these patients were classed as having HGS; where there was unanimous agreement that there was either ‘probably not’ or ‘definitely not’ the presence of an HGS, these were classed as no HGS (without HGS). Wilcoxon’s signed rank was used to test the ability to differentiate cases with and without HGS.

Univariate logistic regression models were used to evaluate the diagnostic performance of MRCP+ metrics in distinguishing between cases with and without HGS. Receiver operating characteristic (ROC) curves were generated, and the area under the curve (AUC) along with 95% confidence intervals was calculated. All statistical analyses and randomizations were conducted using IBM SPSS Statistics for Macintosh, version 22.0 (Armonk, NY, USA: IBM Corp.), with a significance threshold set at *p* < 0.05.

## 3. Results

### 3.1. Patient Demographics and Diagnosis

In this study, N = 28 patients with PSC (mean age 45 ± 14 years, 71% male) met the inclusion criteria. All participants were receiving treatment with ursodeoxycholic acid (UDCA) to manage PSC. Table 1 shows a summary of patient demographics. The radiologists agreed that 39% (11/28) had HGS following the consensus HGS definition. For both intra- and extra-hepatic ducts, between the three independent reads, there was full agreement in at least 21% (6/28) of cases, partial agreement in at least 50% (14/28), and complete disagreement in up to 25% (7/28) of the cases evaluated (Supplementary Table S2). In this study, all MRCP scans were analyzed successfully using MRCP+.

### 3.2. Inter- and Intra-Reader Agreement: Traditional MRCP vs. MRCP+

Intra-reader agreement for assessing the presence of HGS using MRCP alone was high for all three radiologists, with no significant differences between observations for intra- and extra-hepatic ducts (64.3% vs. 70.8%, *p* > 0.05, respectively) from both reads (Individual intra-reader assessments are reported in Supplementary Table S3). A significant difference was observed in the inter-reader agreement between the consulting radiologists in the first session of MRCP reads for HGS detection in intra-hepatic and extra-hepatic ducts (42.9% vs. 66.1%, *p* < 0.01 respectively) (Table 2). The poor inter-reader agreement in HGS identification for intra-hepatic ducts was supported by a low Cohen’s Kappa coefficient value of 0.36 ± 0.12. Moreover, inter-reader agreement for the modified Amsterdam score was low for both intra-hepatic (16.1%) and extra-hepatic ducts (39.3%).

Information provided by MRCP+ reports significantly improved the inter-reader agreement for HGS detection in intra-hepatic ducts compared to the first MRCP-only read (67.9% vs. 42.9%, *p* = 0.02) (Table 2). There was an increase in inter-rater agreement for HGS with the introduction of MRCP+ compared to the first MRCP-only read with (κ = 0.53 ± 0.12 vs. κ = 0.26 ± 0.13, *p* < 0.001, respectively). Furthermore, while the PPA for agreement on HGS presence marginally decreased by 10% for intra-hepatic ducts (MRCP: 66.7% vs. MRCP+: 56.7%, *p* = 0.012), its NPA increased by 31.7% (MRCP: 62.3% vs. MRCP+: 94.0%, *p* < 0.001).

### 3.3. MRCP+ Metrics and High-Grade Stricture Detection

**Figure 3:**
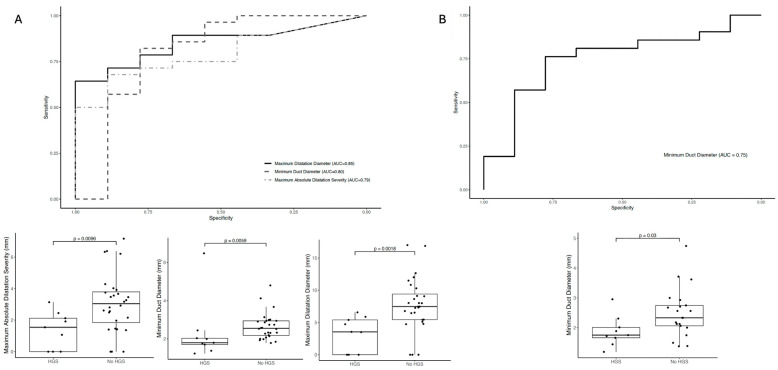
Diagnostic performance of advanced MRCP+ metrics to differentiate between patients with and without (**A**) extra-hepatic HGS and (**B**) intra-hepatic HGS, according to radiologists’ interpretation of MRCP reads. For the boxplots, the lines indicate the median and lower and upper quartiles. Abbreviations: AUC, area under the receiver operating characteristic curve; HGS, high-grade stricture; MRCP+, quantitative magnetic resonance cholangiopancreatography.

MRCP+ metrics stratified patients with and without HGS in extra-hepatic ducts with good diagnostic accuracy (Figure 3). The maximum absolute dilatation severity, minimum duct diameter, and maximum dilatation diameter metrics had the highest diagnostic performance in identifying patients with HGS in extra-hepatic ducts with AUCs of 0.79 (95% CI: 0.64–0.94), 0.80 (95% CI: 0.58–1), and 0.85 (95% CI: 0.73–0.97), respectively. For the identification of HGS in intra-hepatic ducts, the minimum duct diameter metric had the highest diagnostic performance with AUC: 0.75 (95% CI: 0.55–0.95) (Figure 3).

## 4. Discussion

In this study investigating the impact of quantitative MRCP primarily for the detection of high-grade strictures, we identified two key findings. Firstly, radiologists’ confidence in diagnosing high-grade strictures improved significantly with the inclusion of MRCP+ compared to the initial MRCP read alone with regards to intrahepatic strictures. Although there was an improvement in radiologists’ confidence on the second read of MRCP alone, further improvement was noted through the addition of MRCP+. Secondly, when assessing the diagnostic performance of quantitative MRCP metrics to identify HGS, the maximum dilatation diameter had the highest diagnostic performance in identifying HGS in extra-hepatic ducts (AUC: 0.85), whilst the minimum duct diameter metrics had the highest diagnostic performance for identifying HGS in intra-hepatic ducts (AUC: 0.75).

Quantitative MRCP imaging offers a reproducible and objective evaluation of the biliary tree, capturing detailed metrics such as the number, length, and severity of strictures and dilatations, as well as total biliary volume [9]. Using non-contrast MRCP images, MRCP+ generates a 3D model of the biliary system, allowing for regional volumetric analysis of the bile ducts, pancreatic duct, and gallbladder [9]. Recent studies have demonstrated that MRCP+ metrics correlate with surrogate markers of disease severity, including the extent of intrahepatic dilatations [15], and have shown prognostic value in predicting transplant-free survival in patients with PSC [16]. In this study, MRCP+ metrics had very good diagnostic accuracy (AUC ≥ 0.8) for the detection of HGS in intra- and extra-hepatic biliary ducts. The additional information provided by the MRCP+ resulted in a significant, almost double, increase in inter-reader agreement for HGS detection in intra-hepatic ducts compared to the use of traditional MRCP. This significant improvement illustrates the clinical utility that MRCP+ can have in reducing the subjectivity inherent in traditional interpretations, thereby contributing to a more reliable diagnostic framework.

Regarding the real-world implementation of MRCP+ when compared to traditional MRCP, in addition to using the same 3D MRCP acquisition as traditional (standard) 3D MRCP, the MRCP+ imaging protocol increases the resolution of the acquired images via isotropic image acquisition [9]. Furthermore, in conjunction with having a dedicated post-processing method that enhances the visualization of the biliary tree, suppressing the low intensity signal abdominal structures, MRCP+ improves the visualization of small-caliber ducts with automatic and quantification measurements that provide duct diameter, number of ducts, number of strictures, number of dilatations, and biliary tree volume, among other biliary metrics [9]. In addition, unlike AI tools that rely on deep-neural-network classifiers to analyze 3-D MRCP images [17], MRCP+ is fully standardized across scanners and field strengths. It generates a color-coded, anatomically constrained 3-D model of the entire biliary tree along with comprehensive quantitative metrics, enabling longitudinal monitoring of any cholangiopathy, even when applied to historical datasets [9].

Delayed diagnosis, especially in problematic cases with HGS, can result in significant morbidity and mortality [18]. Currently, the diagnosis of high-grade strictures (HGS) in the intra- and extra-hepatic bile ducts is subjective and relies heavily on expert radiologist interpretation, which limits reproducibility and makes it challenging to compare results across different centers. In this study, there was a significantly higher inter-reader agreement for the second read of MRCP. Increased agreement with increased frequency of image review is not a novel finding. However, although potentially better for patient assessment and typically employed during clinical trial central imaging reads, in routine care, double reads of MRCP images are not common nor feasible primarily due to time and resource constraints. Furthermore, since the second read was conducted after the MRCP+ training, there may have been potential bias introduced as a result of the training. Nevertheless, variation in expertise across centers, as observed in this study where there was <30% full agreement and <60% partial agreement between expert hepatobiliary readers, can lead to high incidence of misdiagnoses and unnecessary invasive procedures (such as ERCP), all of which affect disease progression and patient quality of life [7,8]. Whilst traditional diagnostic methods such as ERCP are accurate, they are invasive and are associated with post-procedural complications, which can have considerable health economic costs (~$4452 per patient in 2015 [7]). Hence, as PSC is characterized by multi-focal stricturing and potential malignancy, there is a need for noninvasive, objective, and robust diagnostic tools to support both diagnosis and monitoring and improve efficiency within this patient pathway [19].

While the present study presents an advancement in the field of PSC diagnostics, we must acknowledge some study limitations. Our study only evaluated the impact on decision-making for a small group of experienced readers. It is feasible that there would be a greater impact on decision-making in those with less experience, and this may have yielded greater insight into the impact of MRCP+ on the standardization of diagnosis more widely. Alongside including readers with varying expertise, future studies should also include larger statistically powered sample sizes to achieve broad statistical generalization and validate these findings. These studies should also include a wide variety of cases, such as those with low-grade and high-grade stenosis, to more comprehensively evaluate the effectiveness of MRCP+ clinical practice universally. Nevertheless, our results show a positive impact on expert diagnosis of high-grade strictures with a 1.5-fold increase in inter-reader diagnostic confidence. In this study, we observed an increase in the inter-reader consistency between the first two reads. We conducted the intra-reader consistency assessment over a longer period. Although a 1-to-4-week gap between readings is generally recommended to strike a balance between reducing recall bias and variability, shorter time frames may be desirable to minimize the impact of variability. This was a cross-sectional study, and thus changes in the metrics in relation to clinical outcomes were not investigated. Future longitudinal studies will offer a clearer understanding of how these metrics change over time, enhancing understanding of their utility for disease monitoring, sensitivity to treatment-related changes, and associations with key clinical outcomes. Alongside the assessment of longitudinal changes, these studies should also investigate the value of multiple repeated readings, the prognostic ability of MRCP+ metrics, and the utility of these quantitative metrics to predict adverse outcomes and should compare the performance of MRCP+ metrics to existing markers. In this study, we saw an increase in the agreement specifically for intrahepatic HGS, where treatment is less frequently performed due to technical complexity compared to extrahepatic HGS, which highlighted the impact these metrics can have on decision-making and subsequent patient management. Clinical decision-making is multifactorial and does not rely on the use of one modality [20]. Therefore, alongside looking at prognostic utility, future studies evaluating the feasibility of incorporation of MRCP+ into clinical practice should evaluate the impact of the technology on radiological assessment as part of a clinical decision support study. This will yield the evidence required to support incorporation into practice. Additionally, these evaluations will support health economic evaluations associated with the inclusion of MRCP+ in clinical practice.

In this study, only a small cohort of patients with HGS was evaluated. Future larger studies should evaluate longitudinal changes in the biliary tree using MRCP+ alongside assessment of clinical benefit including symptomatic changes (e.g., pruritus presence, severity, and change). This is especially important as there is a lack of a reference standard for reader results, and over 50% of patients with PSC develop high-grade strictures during the course of the disease [5]. Additionally, patients with cirrhosis or decompensation were excluded from this study. Although we wanted to ensure that the findings were not confounded by the effects of advanced liver disease (e.g., the presence of ascites), future studies must include assessment of these patients to understand better the clinical utility and cost-effectiveness of using MRCP+ as part of the evaluation and monitoring pathway. This inclusion may have the potential to inform more timely planning for liver transplantation for these patients. The ERCP data for the patients involved was not readily available at the time of analysis; thus, the data for those who underwent ERCP were not included as part of this analysis. We acknowledge the limitations this brings to the study, as ERCP can be viewed as a reference standard for the assessment of high-grade stenosis. Nevertheless, HGS were defined using MRCP according to accepted criteria and reporting standards developed by the International Primary Sclerosing Cholangitis Study Group [5]. Future studies should consider inclusion of patients with ERCP data, as this can provide further validation of the utility of MRCP+, in addition to increasing confidence and reduction in the reader variability reported herein.

In conclusion, MRCP+ provides imaging metrics that can improve confidence in the diagnosis of high-grade strictures and may help to reduce inter-reader and intra-reader variability. Quantitative MR metrics had good diagnostic performance to identify radiologist-defined HGS. Given the high rate of adverse clinical outcomes associated with the development of high-grade strictures, accurate, consistent assessment is important, and MRCP+ may offer an objective, non-invasive alternative to support clinicians alongside standardizing biliary tree assessment.

## Figures and Tables

**Table 1:** Patient cohort characteristics. Abbreviations: ALT, alanine aminotransferase; ALP, alkaline phosphatase; AST, aspartate aminotransferase; UDCA, ursodeoxycholic acid.

	Alignment Cases (*n* = 16)	Scans							
		Baseline				Follow-Up			
		All Patients (*n* = 28)	HGS (*n* = 11)	No HGS (*n* = 17)	*p* Value	All Patients (*n* = 28)	HGS (*n* = 11)	No HGS (*n* = 17)	*p* Value
Age (years)	37 ± 18	45 ± 14	45 ± 13	45 ± 16	0.981	46 ± 15	46 ±14	45 ± 16	0.944
Male gender [*n* (%)]	7 (44%)	20 (71%)	8 (73%)	12 (71%)	0.907	20 (71%)	8 (73%)	12 (71%)	0.907
UDCA dose (mg/kg)	2.3 ± 4.2	5.2 ± 5.7	6.8 ± 5.0	4.1 ± 6.1	0.191	5.3 ± 5.8	6.8 ± 5.0	4.0 ± 6.0	0.175
**Liver Biochemistry, Imaging, and Risk Scores**									
Bilirubin (µmol/L)	18 ± 15	14 ± 8	14 ± 8	14 ± 8	0.981	21 ± 40	28 ± 58	16 ± 15	0.991
ALP (U/L)	264 ± 259	253 ± 263	250 ± 153	255 ± 320	0.269	214 ± 164	246 ± 182	187 ±143	0.196
ALT (U/L)	105 ± 114	66 ± 51	71 ± 44	63 ± 57	0.397	74 ± 67	67 ± 47	74 ± 76	0.621
AST (U/L)	81 ± 104	48 ± 29	52 ± 28	45 ± 30	0.359	52 ± 41	50 ± 29	51 ± 46	0.410
Liver stiffness (kPa)	13.2 ± 15.6	9.2 ± 4.3	10.4 ± 5.2	8.4 ± 3.6	0.230	10.5 ± 6.7	12.2 ± 7.7	8.9 ± 5.6	0.115
Mayo risk score	−0.8 ± 1.1	−0.9 ± 0.8	−0.9 ± 0.8	−0.8 ± 0.7	0.654	−0.8 ± 1.0	−0.76 ± 1.0	-0.78 ± 1.0	0.732

**Table 2:** Summary of intra- and inter-reader agreement in the detection of high-grade strictures (HGS) and assessment of disease progression in patients with primary sclerosing cholangitis.

Clinical Assessment	Location	Measurement	Intra-Reader (%)	Inter-Reader		
				MRCP Read 1 (*n* [%])	MRCP Read 2 (*n* [%])	Quantitative MRCP (*n* [%])
High-grade stricture detection	Intrahepatic	Agreement	64.3 ± 9.4	12 (42.9%)	17 (60.7%)	19 (67.9%)
		Confidence	56.2 ± 2.1	14 (50.0%)	15 (54.2%)	18 (64.3%)
	Extrahepatic	Agreement	70.8 ± 6.3	19 (66.1%)	19 (67.9%)	19 (67.9%)
		Confidence	58.3 ± 3.9	16 (55.4%)	17 (60.1%)	17 (59.5%)

## Data Availability

The data and analytical methods used in this study are owned by the study sponsors. Deidentified participant data may be shared with qualified researchers upon request, subject to sponsor approval, investigator support, and completion of a signed data access agreement.

## Supplementary Materials

The following supporting information can be downloaded at: https://www.mdpi.com/article/10.3390/jcm14155530/s1, Table S1: Names of provided metrics within all Quantitative MRCP reports provided to radiologist and calculations of each; Table S2: Summary of the radiologists’ inter-reader agreement in the detection of high-grade strictures (HGS). Full agreement was when all three radiologists agreed, partial agreement indicates when two out of three radiologists agreed, and disagreement was when all radiologists disagreed; Table S3: Summary of the individual radiologist’s intra-reader agreement in the detection of high-grade strictures (HGS).
