# Supplementary material for: Exploring the Role of MRCP+ for Enhancing Detection of High-Grade Strictures in Primary Sclerosing Cholangitis
Source: J Clin Med. 2025 Aug 6;14(15):5530. doi: 10.3390/jcm14155530 (PMC12347560; doi:10.3390/jcm14155530)
Supplement: Supplementary file 1 [file jcm-14-05530-s001.zip › jcm-3769038-supplementary.pdf]

Supplementary Table S1: Names of provided metrics within all Quantitative MRCP reports provided to radiologist and calculations of each.

Name of Advanced Metric	Method of Calculation
**Biliary System Metrics**	
Number of ducts (n)	The number of ducts within whole biliary tree.
Total number of strictures (n)	Number of strictures within whole biliary tree.
Total number of dilatations (n)	Count of number of dilatations within whole biliary tree.
Biliary tree volume (mL)	Total volume of biliary tree.
Gallbladder volume (mL)	Volume of gallbladder.
Total length of ducts (mm)	Sum of all duct lengths measured along the centreline.
Total length of strictures and dilatations (mm)	Sum of all lengths of strictures and dilatations measured along the centreline.
Total length of strictures (mm)	Sum of all stricture lengths measured along the centreline.
Sum of relative score of strictures	Sum of all relative stricture score.
Number of ducts with strictures or dilatations (n)	Total count of stricture and dilatations.
Sum of absolute stricture severity (mm)	Sum of the absolute severity of strictures. Size of decrease (in mm) of duct diameter of stricture site with reference to a local maximum diameter.
**Individual Duct Metrics**	
Duct diameter (median) (mm)	The median diameter of the selected modelled duct.
Maximum duct diameter (mm)	The maximum diameter of the selected modelled duct.
Minimum duct diameter (mm)	The minimum diameter of the selected modelled duct.
Duct diameter IQR (mm)	Interquartile range of selected duct diameter.
Duct length (mm)	Length of selected modelled duct measured along the centreline.
**Stricture and Dilatation Metrics**	
Number of strictures (n)	Stricture counts within a selected duct.
Number of dilatations (n)	Dilatation counts within a selected duct.
Minimum stricture diameter (mm)	Minimum stricture diameter.
Maximum dilatation diameter (mm)	Maximum dilatation diameter.
Maximum stricture length (mm)	Maximum stricture length.
Max dilatation length (mm)	Maximum length of dilatation.
Maximum stricture severity	Percentage decrease of duct diameter of stricture site with reference to a local maximum diameter.
Maximum dilatation severity	Percentage increase of duct diameter of dilatation site with reference to a local minimum diameter.

Supplementary Table S2: Summary of the radiologists' inter-reader agreement in the detection of high-grade strictures (HGS). Full agreement was when all three radiologists agreed, partial agreement indicates when two out of three radiologists agreed, and disagreement was when all radiologists disagreed.

Ducts	Read	Agreement		
		Full Agreement (N [%])	Partial Agreement (N [%])	Disagreement (N [%])
Intrahepatic Ducts	Read 1	6 (21%)	15 (54%)	7 (25%)
	Read 2	7 (25%)	14 (50%)	7 (25%)
	Read 3	7 (25%)	15 (54%)	6 (21%)
Extrahepatic Ducts	Read 1	7 (25%)	15 (54%)	6 (21%)
	Read 2	9 (32%)	17 (61%)	2 (7%)
	Read 3	10 (36%)	15 (54%)	3 (11%)

Supplementary Table S3: Summary of the individual radiologist's intra-reader agreement in the detection of high-grade strictures (HGS).

Clinical assessment	Location	Measurement	Intra-reader		
			**Radiologist 1** (N [%])	**Radiologist 2** (N [%])	**Radiologist 3** (N [%])
High-grade stricture detection	Intrahepatic	Agreement	17 (60.7%)	16 (57.1%)	21 (75.0%)
		Confidence	16 (56.0%)	15 (54.2%)	16 (58.3%)
	Extrahepatic	Agreement	18 (64.3%)	20 (71.4%)	22 (76.8%)
		Confidence	17 (61.9%)	15 (54.2%)	16 (58.9%)
